# Determination of Tiamulin Concentration in Sow Milk and in Sera of Suckling Piglets

**DOI:** 10.3390/molecules28196940

**Published:** 2023-10-05

**Authors:** Piotr Cybulski, Anna Gajda, Magdalena Bilecka, Artur Jabłoński

**Affiliations:** 1 Goodvalley Agro S.A., Dworcowa 25, 77-320 Przechlewo, Poland; 2 Department of Pharmacology and Toxicology, National Veterinary Research Institute, Partyzantow 57, 24-100 Pulawy, Poland; anna.gajda@piwet.pulawy.pl (A.G.); magdalena.bilecka@piwet.pulawy.pl (M.B.); 3 Department of Pathology and Veterinary Diagnostics, Institute of Veterinary Medicine, Warsaw University of Life Sciences, Nowoursynowska 159C, 02-776 Warsaw, Poland

**Keywords:** antibiotics, pleuromutilins, tiamulin, pigs, sows, milk, serum, UHPLC-MS/MS

## Abstract

Although modern analytical methods developed for monitoring antibiotics in several biological matrices are easily available, none of them have been applied to evaluate the transfer of tiamulin into sow milk. Therefore, this work was intended to analyse the concentrations of tiamulin in milk samples collected from lactating sows during and after a treatment consisting of three consecutive intramuscular applications of the antibiotic. The second aim of this investigation was to determine tiamulin concentrations in serum samples obtained from suckling piglets ingesting milk contaminated with the compound. Ultra-high-performance liquid chromatography coupled with tandem mass spectrometry (UHPLC-MS/MS) was used to quantify the analyte in both matrices. Our investigation proved tiamulin was transmitted into the milk of lactating sows. The mean concentration of the antibiotic among samples collected 3 h after administration was 1043 μg/L. The mean level of tiamulin on days 1 and 2 was 876 μg/L and 902 μg/L, respectively. The highest mean concentration of the antibiotic (1061 μg/L) was observed in samples collected on day 3. The mean concentration of the antibiotic in serum samples collected from 3-day-old piglets was 22.2 μg/L. The association between their body weight and serum tiamulin concentration was not statistically significant (*p* = 0.456).

## 1. Introduction

Tiamulin is a semisynthetic derivative of a naturally occurring tricyclic diterpene antibiotic pleuromutilin, a substance produced by the edible mushroom *Clitophilus scyphoides* (formerly named *Pleurotus mutilus*) and some other species of fungi [1,2,3]. The antibiotic was discovered in the 1950s in the USA [1]. A structural formula of tiamulin was elucidated in the 1960s in the UK [4], and afterwards, the drug was patented in the late 1970s in the same country [5]. Tiamulin, alongside valnemulin (the latter was introduced in 1999), is the only pleuromutilin derivate currently accessible on the global veterinary medicine market. Neither active substance is authorised for use in human medicine. Tiamulin is an important antimicrobial agent, which is now commercially available under a broad variety of invented names, either in water-soluble and premixed formulations of tiamulin hydrogen fumarate or as injectable solutions containing purified sesame oil [6]. In general, the mechanism of tiamulin bacteriostatic activity is highly specific and based on the selective inhibition of bacterial translation achieved by binding to the 50S ribosomal subunit [7,8].

Tiamulin is a limited-spectrum antibiotic, with antibacterial properties characterised by its high activity against Gram-positive bacteria, such as staphylococci and streptococci [9,10]; a variety of mycoplasmata [9,10,11,12,13,14,15]; and spirochetes, such as *Brachyspira spp.* [16,17,18]. Moreover, the drug presents selective activity against Gram-negative pathogens, such as *Actinobacillus pleuropneumoniae* [19] and *Lawsonia intracellularis*, among others [20,21]. Therefore, the antibiotic is extensively used in the treatment and metaphylaxis of common pulmonary and gastrointestinal diseases in food-producing animals caused by various bacterial agents, mostly in pigs and to a lesser extent in poultry, with oral medications accounting for the overwhelming majority of sales [22,23,24,25].

According to the available scientific literature, the antibiotic undergoes an extensive metabolic pathway in swine [26,27], but some of the aspects of its clinical pharmacology have not been adequately elucidated so far. Even though the accessible peer-reviewed publications present detailed information about modern analytical methods (including high-performance liquid chromatography—HPLC) developed for monitoring the antibiotic in several biological matrices, such as animal tissues [3,26,28], cow milk [29], honey [30], premixes and medicated feeds [31,32,33], soil [34], liquid manure [35], and swine wastewater [36], these methods have not yet been fully utilised in order to shed light upon the matter of some missing links in modern academic knowledge. Indeed, tiamulin serum and tissue concentrations in pigs following oral and intramuscular administration have been established empirically [3,27,37]; nevertheless, to the best of our knowledge, there is a paucity of information from experimental data on the passage of the antibiotic in question into sow milk.

For all the aforementioned reasons, the main purpose of the research described hereinafter was to analyse the concentrations of tiamulin in milk samples collected from lactating sows during and after a treatment (consisting of three consecutive intramuscular applications of the antibiotic). The second purpose of the investigation was to determine tiamulin concentrations in serum samples obtained from the sows’ progeny.

## 2. Results

### 2.1. Validation of the UHPLC-MS/MS Method

**Figure 1:**
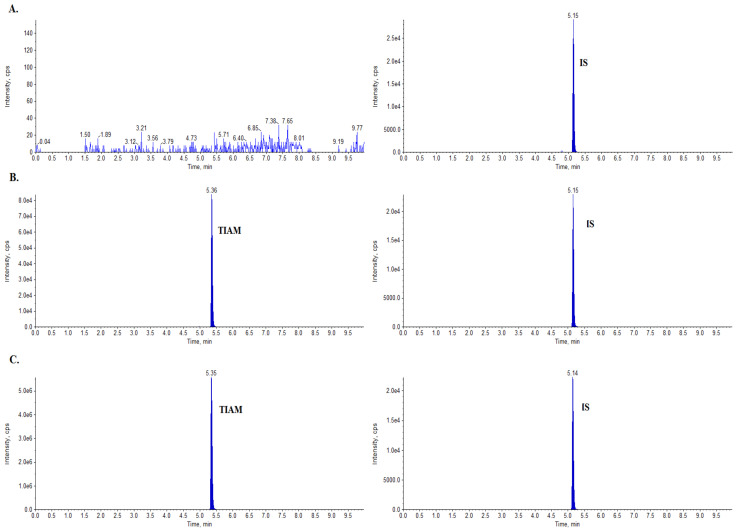
Extracted ion chromatograms (EICs) of (**A**) blank milk sample, (**B**) tiamulin (TIAM) milk sample fortified with 10 µg/L, and (**C**) milk sample obtained 1 day after the intramuscular injection of a veterinary medicinal product with tiamulin at a concentration of 949 µg/L.

The tiamulin concentrations in the collected milk and serum samples were determined using a validated analytical method. The performed method was sensitive, with satisfactory recovery and precision. A good linear response for all matrix-matched calibration curves was observed with r^2^ > 0.999. The linearity for milk was evaluated by preparing two matrix-matched calibration curves in concentration ranges of 1–500 µg/L and 500–2000 µg/L. For serum, a matrix-matched calibration curve was prepared as 1–300 µg/L. No matrix interferences were observed in the retention time of the target analyte. Figure 1 presents the chromatograms of a blank milk sow sample, a sample fortified with tiamulin at a level of 10 µg/L, and a sample determined on the first day after medication. The coefficients of variation, CVs, were less than 10% for repeatability and less than 15% for within-laboratory reproducibility, indicating reproducible and repeatable validation results and the good precision of the presented methods.

The validation results are presented in Table 1. The milk recovery was obtained in the range of 94.0–107%, depending on the fortification level, while for serum, it was calculated as 97.3–106%. The low values of LOQ = 1 μg for milk and serum show the high sensitivity of the method.

The simple sample preparation step of the presented method with the acetonitrile extraction and filtration of samples with the usage of PVDF filters was found to be suitable and labour-efficient for the analysis of tiamulin in many milk samples in a very short time. For the analysis of tiamulin from serum, isolation with 0.5% formic acid and purification with nanosep centrifugal filters were used with satisfactory results. The obtained validation calculations indicated that the developed methods are adequate for the reliable quantification of tiamulin in sow milk and serum.

### 2.2. Detection and Quantification of Tiamulin

**Figure 2:**
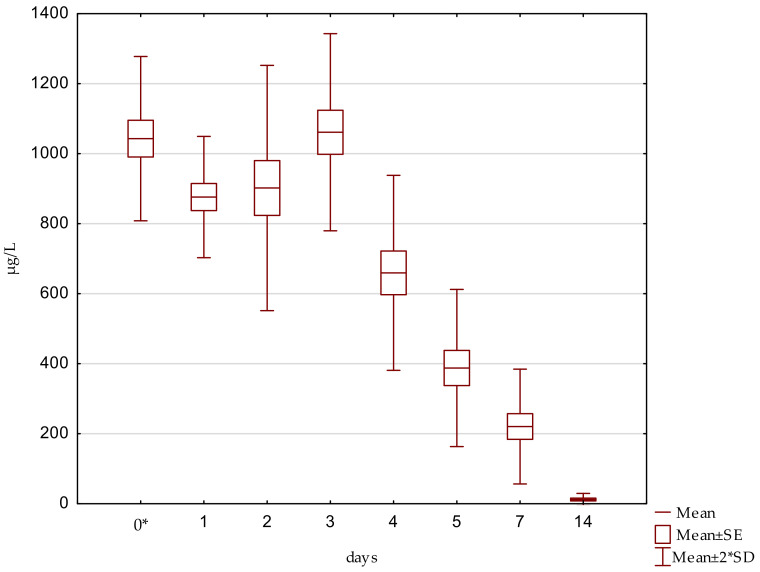
Concentration of tiamulin in sow milk during and after treatment using 3 injectable daily doses of 12 mg of the antibiotic per kg of body weight. * 3 h after the first injection.

Individual and mean tiamulin concentrations in sow milk obtained during the trial are presented in Figure 2. All 40 of the analysed milk samples that had been collected from five sows between 3 h and 14 days following the first intramuscular application of the antibiotic were tiamulin-positive. The mean concentration of the antibiotic among samples collected 3 h after administration was 1043 μg/L, with a slight variation from 922 μg/L to 1190 μg/L. The mean levels of tiamulin on days 1 and 2 were 876 μg/L and 902 μg/L, respectively. The highest mean milk concentration of the antibiotic amid sows included in the investigation, i.e., 1061 μg/L with deviations in individuals varying from 922 μg/L to 1260 μg/L, was observed on day 3. The mean concentrations of the active substance detected in sow milk on days 4, 5, and 7 were significantly lower, reaching 660 μg/L, 388 μg/L, and 221 μg/L, respectively. Tiamulin exhibited the lowest mean concentration in samples collected on day 14 (12 μg/L). The concentrations of tiamulin in all the milk samples collected on day 22 were below the limit of quantification.

The mean body weight among all 15 piglets subjected to the investigation was 1749 g (ranging from 1330 g to 2245 g; SD 265 g). All the serum samples collected from the batch were tiamulin-positive. The mean concentration of the antibiotic was 22.2 μg/L (SD 14.7 μg/L). The values detected in individuals ranged from 1.9 μg/L to 55.6 μg/L. The Pearson correlation indicated that there was a very small negative relationship between the weight of a piglet and its body weight (*r* = 0.209); however, the association between the variables evaluated in the study was not considered significant (*p* = 0.456).

## 3. Discussion

The results presented in this paper clearly demonstrate the effective passage of tiamulin into sow milk after an intramuscular application. An evaluation of samples collected 3 h after a deep intramuscular administration of 12 mg of the antibiotic per kg of body weight resulted in a concentration of 1043 μg/L; therefore, the milk concentration–time profile of the antibiotic after its application to lactating sows can be characterised by a relatively rapid absorption plateau phase and a good distribution throughout the extravascular compartment. Moreover, the injection followed by two redosings administered every 24 h provided a 72-h-long phase marked by a stable level of the active substance in milk. The mean milk concentrations evaluated on days 4 and 5 of the trial were 37.8% and 63.4% lower than the mean value noted on day 3.

As specified in the drug information leaflet, tiamulin reaches its maximum plasma concentration (i.e., 610 μg/L) 4 h post-injection [38]. The duplication of an analysis of plasma concentrations employing a group of sows as a study model was outside the scope of our research; nevertheless, having taken into consideration the official pharmacokinetic data declared by the drug manufacturer, as well as the mean values detected in our investigation, the prior mentioned milk concentration (that had been assessed 1 h before the anticipated occurrence of the corresponding plasma peak) was found to be 1.71 times higher than the most plausible maximum plasma concentration in the sampled sows.

To the best of our knowledge, data on the excretion of tiamulin into sow milk have not been published to date. Thus far, tiamulin milk concentrations were documented only for ruminants, in which the values observed in milk samples were several times higher than those detected in blood [29] (Table 2). Indeed, the highest mean concentration observed in cow milk after the intramuscular application of 10 mg of tiamulin per kg of body weight was 7.5 times higher than the mean peak serum concentration; however, the specimens were collected from culled animals with partially functioning mammary glands. Considerable similarities were observed in ewes and goats receiving the same dosage of the antibiotic.

Theoretical ratios between milk and plasma concentrations of the active substance in lactating sows could have been estimated using the Henderson–Hasselbalch equation [39], although the reliability of the process appears to be severely limited by the lack of a previously proven agreement between concentrations of antibiotics anticipated exclusively on calculations (on the basis of pH partitioning) and pharmacokinetic data (obtained using empirical evidence based on an animal model). In other words, the usefulness of mathematically projected milk-to-serum ratios at equilibrium in swine seems to be dubitable and requires further investigation focused on the fundamental principles of xenobiotics permeability across swine mammary tissue along with additional factors affecting their excretion in milk.

The present study showed that the administration of tiamulin to lactating sows resulted in a low antibiotic concentration in serum specimens collected from suckling piglets on day 3. Moreover, even though all the sampled animals had been provided with milk contaminated with a stable level of tiamulin, the serum concentrations of the drug differed markedly. The relatively high variation in the noted values among single piglets did not significantly correlate with their body weight. The most plausible explanation for this observation is the fluctuating consumption of milk caused by several underlying circumstances that were not investigated in our study, including disruptions of suckling patterns in individuals and factors affecting circadian milk yield together with those influencing milk availability in a specific gland.

Derivatives of pleuromutilin are not authorised to be used in ruminant production; therefore, extrapolation of safety standards set for human consumption into suckling piglets (obviously, taking into account the proportionally high ingestion of tiamulin-contaminated milk combined with a relatively low body weight) would have been achievable only if the veterinary authorities had established the maximum residue limits for the antibiotic in question in cow milk, or in milk from some dairy species other than cattle.

Tiamulin is inactive against *Enterobacteriaceae*; however, low concentrations of the drug have been found in vitro to have a considerable influence on the haemagglutinating properties of K88 and K99 *Escherichia coli*, including strains obtained from diarrhoeic neonatal piglets [40]. It is noteworthy that the lowest concentration tested in the aforementioned research that was found to still remain effective was 3 μg/L, which is approximately three times higher than the mean value obtained from all the samples collected during the first 3 days of our investigation. Nevertheless, concentrations lower than 3 μg/L were not tested in the cited work, so the influence on suckling piglets in our study remains speculative.

Since neonatal diarrhoea is one of the key contributors to increased pre-weaning mortality in swine [41,42,43], further actions elucidating the clinical consequences of tiamulin application to lactating sows, including potential serendipitous effects accompanying the procedure, as well as prospective deterioration caused by alterations in the intestinal microbiota would definitively prove beneficial.

## 4. Materials and Methods

### 4.1. Animal Experiment and Sample Collection

The experiment was carried out in August and September 2022 on a sow farm (8000 DanBred sows) located in Poland. The sows were reared using a weekly farrowing system on a slatted floor under conditions meeting the requirements of Council Directive 2008/120/EC of 18 December 2008, which sets the minimum standards for the protection of pigs, and the sows received regular wheat-based lactation feed in pellet form. The levels of protein, fat, and fibre in the feed were 16.0%, 5.4%, and 4.3%, respectively.

A group of 5 randomly selected multiparous sows was used to evaluate the penetration of tiamulin into sow milk. All the sows were given deep intramuscular injections of tiamulin by a veterinarian at 12 mg of compound per kg of body weight for 3 consecutive days using Tiamowet inj. (active substance in a concentration of 162.2 mg/mL; Vetoquinol Biowet Sp. z o.o., Gorzów Wielkopolski, Poland). The treatment administered within the first day postpartum was part of a program designed for the reduction of the vertical transmission of *Mycoplasma hyopneumoniae*. The withdrawal time declared by the producer was 10 days.

With the purpose of evaluating the milk concentration–time profile of tiamulin in lactating sows after the deep intramuscular application of the antibiotic, milk samples were collected manually by a veterinarian 9 times: just before the drug administration; 3 h after; and then at systematic intervals, i.e., 1, 2, 3, 4, 5, 7, 14, and 22 days after the first injection. Prior to the treatment, all sows had negative results for the presence of the antibiotic in milk. Each 25 mL sample was expressed into a sterile plastic screw-top 100 mL specimen jar, allowed to cool down, and stored at −19 °C until the analysis.

In order to assess the tiamulin concentration in the sera of suckling piglets ingesting milk contaminated with the compound within 3 consecutive days, a total number of 15 randomly selected piglets from 3 litters (5 each born from sows 1, 2, and 3) were chosen as the donors of the venous blood samples. To prove full traceability, all the piglets born from the sows in the trial were ear tagged with an individual sow’s identification number. The blood samples were obtained through the puncture of the cranial vena cava performed by a veterinarian operating disposable 0.7 mm (22 G) needles and a 6 mL sterile vacuum tube system containing a clot activator (MLVacuCol, Medlab-Products Sp. z o.o., Raszyn, Poland). The sera were separated after centrifugation. In pursuit of identification of a correlation between serum drug concentrations in suckling piglets and their body weight, all the sampled piglets were individually weighed immediately after blood collection using a digital scale.

### 4.2. Animal Experiment and Sample Collection

#### 4.2.1. Reagents, Chemicals, and Standards

All organic solvents used were of an analytical grade. The tiamulin fumarate standard and internal standard (IS) of sulfaphenazole were purchased from LGC Standards (Teddington, Middlesex, UK). Acetonitrile was from J.T. Baker (Deventer, the Netherlands). Heptafluorobutyric acid and formic acid were from Sigma-Aldrich (St. Louis, MO, USA). Hydrophilic polyvinylidene fluoride (PVDF) membrane syringe filters, 0.22 μm, were provided by Restek (College, PA, USA). Nanosep centrifugal filters with 0.2 µm of Bio-Inert were from Pall Corporation (New York, NY, USA).

A tiamulin and sulfaphenazole stock standard solution at a concentration of 1000 μg/mL in methanol was kept stable for 6 months by storing at −18 °C in the dark. Working solutions were made in ultrapure water from a diluted stock solution and stored at 4–8 °C for 1 month.

#### 4.2.2. Sample Preparation and UHPLC-MS/MS Analysis

For the extraction of tiamulin from milk samples, an aliquot of 2 mL was deposited into a polypropylene centrifuge tube, and the internal standard was added and briefly vortex-mixed. Extraction was performed using 8 mL of acetonitrile. The samples were vortex-mixed for 10 min and centrifuged at 3500 rpm for 15 min. Next, 6 mL of supernatant was transferred to a glass tube and evaporated to dryness using a nitrogen stream at 45 °C. The dry residue was reconstituted in 0.6 mL of 0.025% HFBA, filtered through a 0.22 mm PVDF filter, transferred to an HPLC vial with a glass insert, and analysed using UHPLC-MS/MS.

For the extraction of tiamulin from serum samples, 400 µL of 0.5% formic acid was deposited into an Eppendorf tube, vortex-mixed, transferred to nanosep tubes with 0.2 µm of Bio-Inert, and centrifuged for 15 min. The obtained extract was poured into a chromatographic glass vial and analysed by UHPLC-MS/MS.

The analysis of tiamulin in milk and serum was carried out using ultra-high-performance liquid chromatography with tandem mass spectrometry (UHPLC-MS/MS). The LC separation was performed using a Shimadzu Nexera X2 UHPLC system (Shimadzu, Japan). For the milk analysis, a Poroshel 120 EC-C18 column, 150 mm × 2.1 mm × 2.7 μm (Agilent Technologies, Santa Clara, CA, USA) with a mobile phase of 0.025% heptafluorobutyric acid in water (solvent A) and acetonitrile (solvent B) was used. The gradient mode was as follows: 0–5.0 min at 5% of solvent B, 5.01–8.00 min after increasing to 80% of solvent B, and 8.01–10.0 min after decreasing to 50% of solvent B. The oven temperature was set at 35 °C with a flow rate of 0.3 mL/min and injection volume of 5 μL. The LC analysis of tiamulin in plasma was carried out using a Zorbax SB-C18 column, 50 mm × 2.1 mm × 1.8 μm (Agilent Technologies, USA) with a mobile phase of 0.025% heptafluorobutyric acid in water (solvent A) and acetonitrile (solvent B). The gradient mode was as follows: 0–4.0 min at 10% of solvent B, 4.01–5.30 min after increasing to 80% of solvent B, and 5.31–7.0 min after decreasing to 10% of solvent B. The oven temperature was set at 35 °C with a flow rate of 0.6 mL/min and injection volume of 2 μL.

The monitored tiamulin and sulfaphenazole precursor → product ion pairs were 494 → 192/118 and 315 → 156, respectively. The established mass spectrometer parameters were curtain gas (N2): 20; nebuliser gas (N2): 60; auxiliary gas: 65; ion spray voltage: 4500 V, temperature: 450 °C. The MS/MS parameters for tiamulin were declustering potential (DP): 128 V; cell exit potential (CXP) for ion 1 and ion 2: 18 and 6 V, respectively; entrance potential (EP): 10 V; and collision energy (CE) for ion 1 and ion 2: 30 and 56 V, respectively. For sulfaphenazole as IS, the values were set at DP: 90 V; CE: 26 V; CXP: 15 V; and EP: 10 V.

#### 4.2.3. Analytical Method Validation

The methods used in this study were validated according to the Commission Regulation (EU) 2021/808 [44]. Validation was carried out for linearity, selectivity, precision (repeatability and within-laboratory reproducibility), and recovery. As an additional step, the limit of quantification (LOQ) was calculated. The lowest validated concentration of the LOQ was established at S/N > 10. The linearity for milk was evaluated by preparing two matrix-matched calibration curves in the concentration ranges of 1–500 µg/L and 500–2000 µg/L. For the serum, the matrix-matched calibration curve was prepared as 1–300 µg/L. Precision was evaluated by calculating the coefficients of variation (CVs, %) at each fortification level. Repeatability and within-laboratory reproducibility were determined via the analysis of 6 milk and 6 serum samples spiked with tiamulin at four concentrations: 1, 5, 10, and 100 µg/L. Within-laboratory reproducibility was established by different operators and days by analysing 3 sets of samples (*n* = 6), while repeatability was determined by the same person on the same day.

Concentrations were calculated in reference to internal standards using a matrix-matched calibration curve. Based on the results obtained in the precision study, the average recovery was studied. The specificity of the method was determined by analysing 20 milk and 20 serum samples.

### 4.3. Statistical Evaluation

The statistical analysis was performed using Microsoft Excel 2019 (Microsoft Corporation, Redmont, WA, USA). A *p* value ≤ 0.05 was considered significant. The Pearson correlation coefficient was used to identify the relationship between the individual serum concentration of tiamulin and the body weight of the tested piglets.

## 5. Conclusions

In the field of veterinary medicine, detailed data on antibiotics transfer into sow milk are very limited. The results obtained in our investigation provide a thorough description of the transmission of tiamulin into sow milk after deep intramuscular injections of the antibiotic. The experiment provides valuable insight into veterinary pharmacology and clearly illustrates the long-term exposure of suckling piglets to relatively low doses of tiamulin; nevertheless, the potential biological effects of contaminated milk ingestion remain obscure and require additional systematic research.

## Figures and Tables

**Table 1:** Validation parameters of the method for the analysis of tiamulin in sow milk and serum.

Analyte	Fortification Level [µg/L]	Repeatability [%] (*n* = 6)	Reproducibility [%] (*n* = 18)	Recovery [%] (*n* = 18)
Tiamulin	Milk			
	1.0	8.8	12.8	94.0
	5.0	5.6	5.1	107
	10 100	9.6 8.4	11.7 12.4	103 105
	Serum			
	1.0	7.9	13.4	97.3
	5.0	6.6	8.1	104
	10 100	9.8 8.0	12.2 11.5	106 102

**Table 2:** Concentrations of tiamulin in milk and blood after an intramuscular application of the antibiotic in different species of food-producing animals.

**Species**	**Animals Tested**	**Tiamulin Dosage**	**Mean Peak Serum Cmax**	**Mean Peak Blood Cmax**	**Milk Cmax to Blood Cmax**	**Ref.**
	***n***	*mg/kg*	*μg/L*	*μg/L*	*ratio*	
Goats	23	10	560	2500	4.46	[29]
Ewes	8	10	640	7500	11.72	[29]
Sows	5	12	610	1043	1.72	[this study]

## Data Availability

Data are contained within the article.

## Sample Availability

Not available.
